# Prevalence and determinants of unmet need for contraception among women in low and high-priority segments for family planning demand generation in Nigeria

**DOI:** 10.1186/s13690-022-00997-x

**Published:** 2022-11-21

**Authors:** Bola Lukman Solanke, Olufemi Mayowa Adetutu, Semiu Adebayo Rahman, Daniel Alabi Soladoye, Michael Olumide Owoeye

**Affiliations:** 1 Department of Demography and Social Statistics, Obafemi Awolowo University, Ile-Ife, Nigeria; 2 Department of Sociology, Bowen University, Iwo, Nigeria

**Keywords:** Unmet need for contraception, Family planning demand generation, Reproductive health, Women, Nigeria

## Abstract

**Background:**

Studies have identified various determinants of unmet need for contraception. These determinants cut across individual, household, community, and health facility levels. Despite this evidence, there remains a lack of information regarding differentials in the prevalence and determinants of unmet need for contraception among women in the low-priority segments (such as women of advanced reproductive age and women living with disabilities) and high-priority segments (such as adolescents, young adults, and unmarried women) for family planning demand generation, hence this study.

**Methods:**

The study design is cross-sectional. The study analyzed merged data from the individual, and persons recode of the 2018 Nigeria Demographic and Health Survey (NDHS). The samples analyzed are 5,147 women in the high-priority segment and 7,536 women in the low-priority segment. The outcome variable in the study was unmet need for contraception. The explanatory variables were selected at the individual, household, community, and facility levels. Statistical analyzes were performed using Stata 14. Three multilevel mixed-effects regression models were fitted. Model 1 was the empty model, while Model 2 included the sets of individual, household, and community variables. Model 3 controlled for the facility-level variables.

**Results:**

Findings show a higher prevalence of unmet need for contraception among women in the family planning low-priority segment compared to women in the family planning high-priority segment. Religion and desired family size were the two individual characteristics that significantly predicted the unmet need for contraception among women in the two segments. Sexual autonomy was the only household characteristic that predicted unmet need for contraception in both segments. There were differences in the community characteristics that predicted unmet need for contraception among women in the two segments.

**Conclusion:**

The prevalence of unmet need for contraception is not the same among women in low and high family planning priority segments. The determinants also differ among women in the two segments. Though, women in different family planning segments have the same contraceptive needs of avoiding pregnancy when not needed, however, getting the needs satisfied requires that existing programs be further examined to develop initiatives that will resonate with each segment of reproductive-age women.

## Plain Language Summary

Existing studies have identified diverse predictors of unmet need for contraception. These predictors cut across individual, household, community, and health facility levels. Despite this evidence, there remains a lack of information regarding differentials in the prevalence and predictors of unmet need for contraception among women in the low-priority segments (such as women of advanced reproductive age and women living with disabilities) and high-priority segment (such as adolescents, young adults, and unmarried women) for family planning demand generation, hence this study. Based on a cross-sectional design, merged data from the individual, and persons recode of the 2018 Nigeria Demographic and Health Survey (NDHS) were analyzed. The outcome variable was unmet need for contraception. The explanatory variables were selected at the individual, household, community, and facility levels. Statistical analyzes were performed using Stata version 14. Three multilevel mixed-effects regression models were estimated. Findings show a higher prevalence of unmet need for contraception among women in the family planning low-priority segment compared to women in the family planning high-priority segment. Religion and desired family size were the two individual characteristics that significantly predicted the unmet need for contraception among women in the two segments. Sexual autonomy was the only household characteristic that predicted the unmet need for contraception in both segments. There were differences in the community characteristics that predicted the unmet need for contraception among women in the two segments. The prevalence of unmet need for contraception is not the same among women in low and high family planning priority segments. The predictors also differ among women in the two segments. Though, women in different family planning segments have the same contraceptive needs of avoiding pregnancy when not needed, however, getting the needs satisfied requires that existing programs be further examined to develop initiatives that will resonate with each segment of reproductive-age women.

## Background

In most developing countries particularly countries in sub-Saharan Africa, continued rapid population growth may undermine reaping the demographic dividends. It may also aggravate diverse environmental problems such as global warming, air pollution, water pollution, and deforestation [1, 2]. Thus, in most developing countries, the implementation of voluntary national family planning programs has been adopted as one of the critical strategies to reduce population growth [3, 4], and to enhance reaping the demographic dividends [5]. The programs seek to reduce the level of unintended pregnancies and to eliminate unsatisfied demand for modern contraception among different segments of men and women. This onerous goal requires that existing family planning programs be tailored to the needs of various segments of people for optimal family planning demand generation. In the Nigerian context, adolescents, young adults, and unmarried people are considered a high-priority segment for family planning demand generation [6]. In contrast, some special population groups, such as women in the advanced reproductive age group [7–10] or women living with disabilities may be considered low-priority segments because they are usually underserved by sexual and reproductive health programs in Nigeria [11] and many other countries [12–18].

Regardless of the segments, substantial proportions of women in Nigeria and other developing countries are still not using a method. However, most of them are fertile, sexually active, and have a desire to avoid pregnancy [19]. Such women have been described as having an unmet need for contraception [20]. Across the world, more than 10% of reproductive-age women are estimated to have an unmet need for contraception. This estimate soared to 20% in Africa [21]. In Nigeria, current estimates of unmet need for contraception show that 12% and 7% of currently married women in the country had an unmet need for spacing and limiting, respectively [22]. Different levels of a high prevalence of unmet need for contraception have also been reported in many other developing countries [23–28]. The continued high prevalence not only elevates the risk of both unintended pregnancies and unsafe abortion among women [29–31] but also does not promote the attainment of universal access to reproductive healthcare services, which is an important 2030 agenda for sustainable development.

Efforts to reduce the prevalence across developing countries have led to several studies [26, 27, 32–36] to identify its basic determinants and their family planning programmatic implications. Identified determinants include individual socio-demographic characteristics such as age [36, 37], religion [38], number of living children [33, 39], parity and maternal education [40, 41], employment status [24], age at first marriage [41], and fertility desire [42, 43]. The determinants also include household characteristics such as the sex of the household head [36], household wealth [44], spousal living arrangements [45], and intimate partner violence [46]. In addition, studies have established that community characteristics are important determinants of the unmet need for contraception. Such characteristics include community education [33, 41], community economic prosperity [47], and geographic region [48].

Despite this evidence, there remains a lack of information regarding differentials in the prevalence and determinants of unmet need for contraception among women in low and high-priority segments for family planning demand generation. This may limit the understanding of family planning initiatives that will resonate with specific segments of reproductive-age women. This study intends to fill the knowledge gap. Findings will provide additional information about the determinants of unmet need for contraception across different segments of women, which may help reduce normative barriers to contraceptive use. Also, findings will provide further inputs for the demand generation and behavior change communication strategy of the current 2020–2024 Family Planning Blue Print in Nigeria [49], as well as the 2021 revised national policy on population for sustainable development [50], which seeks among other objectives to enhance the demographic transition to low fertility level in the country.

The study’s objective was to examine differences in the prevalence and determinants of unmet need for contraception among women in low and high-priority segments for family planning demand generation in Nigeria. The study was guided by two research questions: is the prevalence of unmet need for contraception the same among women in high and low family planning priority segments; and, do the determinants of unmet need for contraception differ by family planning priority segments? The socio-ecological model [51] provided the analytical framework of the study. This model is built on two propositions. On the one hand, human behavior affects and is equally affected by multiple levels of influence, such as peer groups, community, and societal structures. On the other hand, individual behavior shapes the social environment, which in turn shapes individual behavior. This model has been widely applied in the analysis of contraceptive use and unmet need for contraception [9, 47, 52–54] because of increasing evidence that improving access to contraceptive information and services works better when they recognize influences at the individual, community, and structural levels [55].

## Methods

### Study design

The study design is basically cross-sectional with the sole purpose of determining differences in the prevalence and determinants of unmet need for contraception among two groups of women. The design was executed in two stages. Firstly, the reported prevalence of unmet need for contraception among the two groups of women was tested for statistical difference. Secondly, based on the statistical difference, separate regression models were fitted for the two groups of women. The significant determinants were then compared. These procedures provided answers to the two research questions.

### Data and sample

This study analyzed data extracted from the 2018 Nigeria Demographic and Health Survey (NDHS). The NDHS is an offshoot of the Demographic and Health Survey (DHS) program, which is being funded by the United States Agency for International Development (USAID) and implemented across developing countries by the Inner-City Fund (ICF) to assist developing countries in collecting reliable and policy-relevant data [56]. The executing agency for the conduct of the 2018 NDHS is the National Population Commission. The methodology of the 2018 NDHS is available in the public domain (https://dhsprogram.com/pubs/pdf/FR359/FR359.pdf). Though the current study focused on women of reproductive age the dataset from individual recodes did not contain information about women living with disabilities. We thus merged data from the individual, and persons recode, which had information about people living with disabilities. The disability domains included in the survey are difficulties in seeing, hearing, communicating, remembering, or walking.

In line with a recent submission that unmet need for contraception is more appropriately defined when it captures currently married or cohabiting women, who are sexually active, not using a method of contraception, but fertile, and having no desire for an additional child [19], we developed inclusion/exclusion criteria. This definition is appropriate because it reduces the overestimation of the unmet need for contraception. The study thus included only women who were currently married, sexually active in the last one month prior to the survey, not using a method of contraception, fertile, and having no desire for an additional child. In addition, the study excluded from analysis women aged 25 to 34 years. In the data cleaning process, we also excluded women who did not know their fertility desire and women who reported traditional religions due to their small proportions. Following their exclusion, the population was divided into two groups of women. The first group was women aged 15–24 or unmarried. This group was referred to as the high-priority segment for family planning demand generation in line with the family planning blueprint in Nigeria [6]. The group consisted of 5,147 women. The second group was women aged 35–49 or who have at least one type of disability. This group was referred to as the low-priority segment for family planning demand generation in line with evidence of being underserved by existing strategies [8, 11, 12, 15–18]. The group consisted of 7,536 women. The samples were weighted by the DHS weighting factors.

### Study variables

The outcome variable in the study was unmet need for contraception. This was measured as a binary response. Women have an unmet need for contraception if they are sexually active, fecund, currently married or cohabiting, and desire to either postpone their subsequent pregnancy for at least two years or limit childbirth but not using contraception [19]. Otherwise, they do not have an unmet need for contraception. Though few studies have measured the unmet need for contraception as a multinomial response [23, 28], more studies [48, 26, 40, 41, 36] have adopted the binary option. The measure adopted in the study is, therefore, consistent with measures in many existing studies.

The selection of the explanatory variables was guided by empirical evidence and the socio-ecological model. Variables were thus selected at the individual, household, community, and facility levels. However, variables peculiar to only one group of women were not selected. At the individual level, the selected variables are education (none, primary, secondary, or higher), work status (employed or unemployed), religion (Islam or Christianity), knowledge of the fertile period (correct or incorrect), desired family size (small or large), media exposure (low, moderate or high), and pregnancy termination (ever or never experienced). Knowledge of the fertile period was measured in line with the 2018 NDHS which described correct knowledge as ‘halfway between two menstrual periods’ [22]. Small or large family size was measured as having four or fewer children or five or more children. This was based on evidence that **a** substantial proportion of Nigerian women have five or more children [57–59]. Media exposure was derived from the frequencies of reading newspapers, listening to the radio or watching television. We created a composite score of nine, with one to three indicating low exposure, four to six indicating moderate exposure, and seven to nine indicating high exposure.

At the household level, three variables, namely, the sex of the household head (male or female), household wealth quintile (poorest, poorer, middle, richer, or richest), and sexual autonomy (yes, if a woman can refuse partner sex, ask a partner to use a condom or no, if otherwise). This variable was included because it elevates women’s exposure to unintended pregnancies and other adverse sexual health outcomes, such as infection with sexually transmitted diseases [60]. At the community level, the selected variables are type of place of residence (urban or rural), geographic region (Northern or Southern), community literacy level, community justification of gender-based violence, community economic prosperity, and proportion ever used contraception in the community. These variables, with the exclusion of place of residence and geographic region, were derived through the aggregation of individual responses at the cluster level. The derived variables were then partitioned into low, medium or high.

Community literacy indicates the proportion of women in the community who can read or write. Community justification of gender-based violence indicates the proportion of women who justified wife-beating if women go out without permission, neglect children, argue with their partner, refuse sex or burn food. Community economic prosperity indicates the proportion of women in the richest household group. These variables have been examined in many recent studies [24, 33, 37, 47]. Lastly, three variables at the facility level were selected as control variables. These are visitation to a health facility in the last twelve months (yes or no), visitation by family planning workers (not visited or visited), and health insurance (not enrolled or enrolled). These variables are included because of their potential to either provide family planning counseling or enhance access to modern contraceptives and have also been examined in relevant existing studies [37, 41, 61, 62].

### Statistical analyzes

Statistical analyzes were performed at three levels using Stata 14 [63]. The first level of analysis described the socio-demographic characteristics of the sample using frequency distribution and percentages. At the second level of analysis, the outcome and explanatory variables were cross-tabulated to show variations in the outcome variable in relation to variations in the explanatory variables. A Variance Inflation Factor (VIF) was performed to assess the extent of multicollinearity among the variables. This assessment was based on the established principle that any variable showing a VIF score of 10 or more may mislead the estimation of the regression coefficients, and that such variable should not be included in further analysis [64]. We did not apply any of the classical method for variable selection because the interest of the study was not risk factor modelling [65] but predicting unmet need for contraception. However, to ensure adequate model-building, a crude regression model using an unadjusted Odds Ratio (uOR) was fitted for the selection of variables to be included in further analysis. Statistical significance was set at *p* < 0.025. A variable is excluded if it shows no significance in the two segments. This method has been applied in many similar existing studies [36, 41].

Further analysis involved fitting multilevel mixed-effects regression models. This method of analysis was suitable for the study because of the multiple level of influence being examined in the study [66]. The mixed-effects model consisted of fixed effects estimated by the adjusted Odds Ratio (aOR) with a 95% confidence interval and random effects estimated by the Intra-Cluster Correlation Coefficient (ICC). Three models using the Stata xtmelogic command were fitted to examine the effects on the unmet need for contraception. Model 1 was the empty model. This model was without the explanatory variables and was fitted to assess variations in the unmet need for contraception across the communities. Model 2 included the sets of individual, household, and community variables. This was a two-level model, with individuals and households (level 1) nested in the communities (level 2). Model 3 was the full model, which controlled for the facility-level variables. Model adequacy was determined using of Akaike Information Criterion (AIC). Statistical significance was set at *p* < 0.05.

## Results

### Univariate results

Table 1 presents the socio-demographic profile of respondents. The prevalence of unmet need for contraception was higher among women in the low-priority group than women in the high-priority group (22.8% vs. 18.6%). Educational attainments were similar among women in the two groups. However, the proportion of women without formal education was higher in the high-priority group. In contrast, employment was higher among women in the low-priority group. Muslim women were more dominant in the high-priority group (72.0%) compared to Muslim women in the low-priority group (54.4%). Across the two groups, most of the women had incorrect knowledge of the fertile period. Likewise, most of the women in the two groups desired large family size. Women in the low group had improved media exposure. The majority of respondents had never experienced pregnancy termination. In the majority of the households, males were the heads of households. In the high-priority group, women in lower household wealth groups were dominant in the sample, while the low-priority group had more women in the higher household wealth groups. In the high-priority group, slightly less than two-fifths (39.0%) of respondents were sexually autonomous compared to more than half (55.2%) of respondents in the other group.

**Table 1:** Socio-demographic characteristics of respondents

Characteristic	Low-Priority	High-Priority	Characteristic	Low-Priority	High-Priority
	**Number (%)**	**Number (%)**		**Number (%)**	**Number (%)**
**Unmet need for contraception**			**Sexual autonomy**		
No unmet need	5,816 (77.2)	4,191 (81.4)	No	3,378 (44.8)	3,138 (61.0)
Unmet need	1,720 (22.8)	957 (18.6)	Yes	4,158 (55.2)	2,010 (39.0
**Education**			**Place of residence**		
None	3,316 (44.0)	2,526 (49.1)	Urban	3,370 (44.7)	1,514 (29.4)
Primary	1,325 (17.6)	607 (11.8)	Rural	4,166 (55.3)	3,634 (70.6)
Secondary	1,952 (25.9)	1,767 (34.3)	**Geographic region**		
Higher	943 (12.5)	247 (4.8)	Northern	4,440 (58.9)	4,041 (78.5)
**Work status**			Southern	3,096 (41.1)	1,107 (21.5)
Unemployed	1,390 (18.4)	2,543 (49.4)	**Community literacy level**		
Employed	6,146 (81.6)	2,605 (50.6)	Low	2,054 (27.3)	2,068 (40.1)
**Religion**			Medium	2,624 (34.8)	1,902 (37.0)
Christianity	3,438 (45.6)	1,441 (28.0)	High	2,858 (37.9)	1,178 (22.9)
Islam	4,097 (54.4)	3,707 (72.0)	**Community justification of gender-based violence**		
**Knowledge of the fertile period**			Low	2,940 (39.0)	1,509 (29.3)
Correct	2,069 (27.5)	1,070 (20.8)	Medium	2,552 (33.9)	1,624 (31.5)
Inaccurate	5,467 (72.5)	4,078 (79.2)	High	2,044 (27.1)	2,015 (39.2)
**Desired family size**			**Proportion ever used contraceptive in community**		
Small	2,142 (28.4)	1,360 (26.4)	Low	2,169 (28.8)	2,255 (43.8)
Large	5,394 (71.6)	3,788 (73.6)	Medium	2,370 (31.4)	1,736 (33.7)
**Media exposure**			High	2,997 (39.8)	1,157 (22.5)
Low	2,374 (31.5)	2,311 (44.9)	**Community economic prosperity**		
Moderate	3,387 (44.9)	2,139 (41.6)	Low	4,046 (53.7)	3,712 (72.1)
High	1,775 (23.6)	698 (13.5)	Medium	284 (3.8)	147 (2.9)
**Pregnancy termination**			High	3,206 (42.5)	1,289 (25.0)
Never experienced	6,140 (81.5)	4,634 (90.0)	**Visitation to health facility in last 12 months**		
Ever experienced	1,395 (18.5)	514 (10.0)	No	4,492 (59.6)	2,920 (56.7)
**Sex of head of household**			Yes	3,044 (40.4)	2,228 (43.3)
Male	6,937 (92.1)	4,694 (91.2)	**Visitation by family planning worker**		
Female	598 (7.9)	454 (8.8)	Not visited	6,416 (85.1)	4,467 (86.8)
**Household wealth quantile**			Visited	1,120 (14.9)	681 (13.2)
Poorest	1,491 (19.8)	1,282 (24.9)	**Health insurance**		
Poorer	1,420 (18.8)	1,401 (27.2)	Not enrolled	7,243 (96.1)	5,093 (98.9)
Middle	1,283 (17.0)	1,057 (20.5)	Enrolled	293 (3.9)	55 (1.1)
Richer	1,513 (20.1)	870 (16.9)			
Richest	1,830 (24.3)	537 (10.4)			
**Total**	7,536 (100.0)	5,148 (100.0)	**Total**	7,536 (100.0)	5,148 (100.0)

Source: Authors’ analyses based on merged data of individual and persons recodes of 2018 NDHS

More women in the low-priority group reside in urban areas than urban dwellers in the high-priority group (44.7% vs. 29.4%). In both groups, Northern women were dominant in the sample. A higher proportion of women in the low-priority group resides in communities with high literacy levels. But in the high-priority group, a higher proportion of women resides in communities with low literacy level. In contrast, a higher proportion of women in the high-priority group (39.2%) live in communities with a high justification of gender-based violence compared with the proportion (27.1%) in the other group of women. Women in low-priority group reside more in communities with a higher proportion of women who have ever used contraception. Likewise, women in the low-priority group lives more in communities with a high level of economic prosperity. More women in the high-priority group compared with the low-priority group, visited a health facility in the last twelve months preceding the survey. But similar proportions of women in the two groups were visited by family planning workers (14.9% vs. 13.2%). Health insurance enrollment was very poor among the two groups of women.

### Bivariate results

Table 2 presents the prevalence of unmet need for contraception by the socio-demographic characteristics of the respondents. The unmet need for contraception was higher among women who attained primary education in the low-priority group but higher among women who achieved higher education in the other group. The level of unmet need for contraception was higher among employed women in the high-priority group, but a similar level was reported by women in the low-priority group. Christian women had a higher prevalence of unmet need for contraception in the high-priority group, but the opposite was observed among the other women. Women with incorrect knowledge of the fertile period reported a higher level of unmet need for contraception in the low-priority group compared to women in the high-priority group who reported otherwise. Likewise, unmet need for contraception was higher among women who desired large family size in the low-priority group compared to a higher level of unmet need for contraception among women who wanted a small family size in the high-priority group. Media exposure relates inconsistently with the unmet need for contraception in the two groups. In the low-priority group, the level of unmet need for contraception declined consistently as media exposure improves. But in the other group, the level of unmet need for contraception increased always as media exposure improves. In both groups, the unmet need for contraception was higher among women who had never experienced pregnancy termination.

**Table 2:** Prevalence (%) of unmet need for contraception by socio-demographic characteristics

Characteristic	Low-Priority Segment		High-Priority Segment		Characteristic	Low-Priority Segment		High-Priority Segment	
	**Prevalence**	**uOR**	**Prevalence**	**uOR**		**Prevalence**	**uOR**	**Prevalence**	**uOR**
**Education**					**Household wealth quintile**				
None ^RC^	24.1	-	9.7	-	Poorest ^RC^	23.3	-	11.1	-
Primary	25.7	1.05	15.0	1.76*	Poorer	25.9	1.11	12.7	1.21
Secondary	20.9	0.81*	29.8	3.85*	Middle	24.5	0.99	20.6	1.97*
Higher	18.3	0.71*	37.5*	4.22*	Richer	21.8	0.87	27.5	2.63*
**Work status**					Richest	19.7	0.76	33.4	3.47*
Unemployed ^RC^	22.8	-	16.6	-	**Place of residence**				
Employed	22.8	0.99	20.5	1.38*	Urban ^RC^	21.0	-	26.8	-
**Religion**					Rural	24.0	1.22*	15.2	0.57*
Christianity ^RC^	20.7	-	32.3	-	**Geographic region**				
Islam	24.6	1.17*	13.2	0.29*	Northern ^RC^	24.6	-	13.2	-
**Knowledge of fertile period**					Southern	20.2	0.84*	38.3	3.53*
Correct ^RC^	21.1	-	19.6	-	**Community literacy level**				
Incorrect	23.5	1.14*	18.3	0.90	Low ^RC^	23.0	-	11.2	-
**Desired family size**					Middle	25.5	1.15	17.7	1.76*
Small ^RC^	19.6	-	31.4	-	High	20.2	0.82*	32.9	3.68*
Large	24.1	1.24*	14.0	0.37*	**Community justification of gender-based violence**				
**Media exposure**					Low ^RC^	21.6	-	21.3	-
Low ^RC^	24.5	-	13.1	-	Middle	22.5	1.19*	22.4	1.28*
Moderate	22.3	0.85*	20.4	1.61*	High	24.9	1.25*	13.4	0.70*
High	21.4	0.84*	31.2	2.58*	**Proportion ever used contraceptive in community**				
**Pregnancy termination**					Low ^RC^	23.7	-	10.5	-
Never ^RC^	22.9	-	19.5	-	Middle	26.3	1.10	21.7	1.95*
Ever	22.3	0.95	10.5	0.54*	High	19.3	0.80*	29.5	2.76*
**Sex of Head of household**					**Community economic prosperity**				
Male ^RC^	22.7	-	16.5	-	Low ^RC^	23.8	-	14.0	-
Female	24.3	1.07*	40.2	3.62*	Middle	26.0	0.97	17.9	1.50
**Sexual autonomy**					High	21.3	0.86*	31.7	2.40*
No ^RC^	22.0	-	20.4	-					
Yes	24.1	0.90	15.7	0.68*					

*RC* Reference category, *p < 0.025, uOR (unadjusted odds ratio)

Unmet need for contraception was higher among women living in female-headed households in the two groups. The prevalence of unmet need for contraception was inconsistent as household wealth improved among women in the low-priority group, but the prevalence increased progressively as household wealth improved in the high-priority group. Similarly, the prevalence was higher among urban dwellers and Southern women in the high-priority group but higher among rural dwellers and Northern women in the low-priority group. The prevalence of unmet need for contraception increases consistently with variations in community literacy level, the proportion of ever-used contraception in the community, and community economic prosperity in the high-priority group. It, however, showed inconsistent prevalence in the low-priority group. In contrast, the prevalence of unmet need for contraception increases consistently as community justification of gender-based violence varies in the low-priority group, but shows inconsistent prevalence as community justification of gender-based violence varies in the high-priority group.

### Fixed and random effects on unmet need for contraception in low-priority segment

Table 3 presents the fixed and random effects on unmet need for contraception in both the low and high-priority segments. In the empty model (Model 1) based on the ICC, the variations of unmet need for contraception were low (not shown). Also, the result of the AIC indicated that the full model had the best goodness of fit. In Model 2, the individual characteristics that significantly predicted an unmet need for contraception are work status, religion, desired family size, and pregnancy termination. At the household level, only sexual autonomy was the significant predictive factor. At the community level, place of residence, geographic region, and community justification of gender-based violence significantly influence unmet need for contraception. The ICC (ICC = 5.4%) indicated that variations in unmet need for contraception could be attributed more to individual factors than the community factors. The inclusion of the control variables in Model 3 slightly causes changes in the fixed effects on the unmet need for contraception. In the model, employed women were less likely to have an unmet need for contraception than unemployed women (aOR = 0.27, 95% CI: 0.26–0.28). Likewise, Muslim women had lower odds of unmet need for contraception than Christian women (aOR = 0.84, 95% CI: 0.74–0.96). Women who wanted large family sizes were more likely to have an unmet need for contraception than women who wanted small family sizes (aOR = 1.26, 95% CI: 1.10–1.45). Sexually autonomous women were less likely to have an unmet need for contraception than those not sexually autonomous (aOR = 0.53, 95% CI: 0.31–0.90).

**Table 3:** Fixed and random effects on unmet need for contraception in low and high-priority segments

Characteristic predicting unmet contraceptive need	Low-Priority Segment				High-Priority Segment			
	**Model 2**		**Model 3**		**Model 2**		**Model 3**	
	**aOR**	**95% CI**	**aOR**	**95% CI**	**aOR**	**95% CI**	**aOR**	**95% CI**
**Education**								
None ^RC^	-	-	-	-	-	-	-	-
Primary	1.08	0.92–1.28	1.14	0.94–1.38	1.30	0.96–1.77	1.31	0.97–1.77
Secondary	1.22	1.03–1.44	0.96	0.77–1.19	2.13**	1.56–2.91	2.11**	1.61–2.78
Higher	0.98	0.77–1.26	0.87	0.65–1.17	1.84*	1.15–2.94	1.82	1.17–2.83
**Work status**								
Unemployed ^RC^	-	-	-	-	-	-	-	-
Employed	0.87*	0.77–0.98	0.27**	0.26–0.28	1.23**	1.12–1.35	1.07	0.90–1.27
**Religion**								
Christianity ^RC^	-	-	-	-	-	-	-	-
Islam	0.83*	0.71–0.97	0.84*	0.74–0.96	0.60**	0.45–0.79	0.61**	0.48–0.79
**Knowledge of fertile period**								
Correct ^RC^	-	-	-	-	-	-	-	-
Incorrect	1.08	0.96–1.22	1.20	0.96–1.27	1.16	0.94–1.43	1.12	0.91–1.37
**Desired family size**								
Small ^RC^	-	-	-	-	-	-	-	-
Large	1.23**	1.12–1.35	1.26**	1.10–1.45	0.66**	0.54–0.83	0.69**	0.56–0.84
**Media exposure**								
Low ^RC^	-	-	-	-	-	-	-	-
Moderate	0.94	0.81–1.10	0.93	0.80–1.09	0.76*	0.69–0.84	0.69*	0.64–0.75
High	1.07	0.86–1.32	1.05	0.85–1.30	0.35*	0.32–0.38	0.35*	0.33–0.37
**Pregnancy termination**								
Never ^RC^	-	-	-	-	-	-	-	-
Ever	0.86*	0.75–0.99	0.95	0.87–1.03	0.55*	0.40–0.78	0.57**	0.42–0.78
**Sex of head of household**								
Male ^RC^	-	-	-	-	-	-	-	-
Female	1.17	0.94–1.47	1.18	0.94–1.48	1.12	0.92–1.35	1.03	0.85–1.25
**Sexual autonomy**								
No ^RC^	-	-	-	-	-	-	-	-
Yes	0.73**	0.65–0.81	0.53*	0.31–0.90	0.59**	0.49–0.72	0.63**	0.52–0.75
**Household wealth quintile**								
Poorest ^RC^	-	-	-	-	-	-	-	-
Poorer	1.10	0.90–1.34	1.11	0.91–1.35	0.95	0.73–1.24	0.95	0.73–1.23
Middle	0.97	0.77–1.21	0.97	0.77–1.22	1.11	0.81–1.51	1.10	0.81–1.49
Richer	0.85	0.65–1.11	0.86	0.65–1.12	1.03	0.72–1.49	1.02	0.72–1.46
Richest	0.80	0.58–1.11	0.80	0.58–1.11	1.19	0.75–1.88	1.18	0.76–1.84
**Place of residence**								
Urban ^RC^	-	-	-	-	-	-	-	-
Rural	1.81**	1.58–2.07	1.42*	1.16–1.72	0.87*	0.77–0.98	0.84*	0.78–0.92
**Geographic region**								
Northern ^RC^	-	-	-	-	-	-	-	-
Southern	0.91*	0.84-1.00	0.71**	0.62–0.81	1.54*	1.14–2.08	1.45*	1.09–1.92
**Community literacy level**								
Low ^RC^	-	-	-	-	-	-	-	-
Middle	1.17	0.97–1.42	1.62	0.97–1.41	1.12	0.85–1.48	1.12	0.85–1.47
High	1.00	0.77–1.29	0.99	0.77–1.28	1.31	0.91–1.90	1.29	0.90–1.85
**Community justification of gender-based violence**								
Low ^RC^	-	-	-	-	-	-	-	-
Middle	1.14*	1.00-1.30	1.32**	1.15–1.50	1.33*	1.04–1.70	1.28*	1.02–1.62
High	1.81**	1.58–2.07	2.44**	2.20–2.71	1.39*	1.27–1.52	1.46*	1.31–1.62
**Proportion ever used contraceptives in community**								
Low ^RC^	-	-	-	-	-	-	-	-
Middle	1.10	0.92–1.32	1.10	0.92–1.31	1.30*	1.01–1.67	1.29*	1.01–1.65
High	0.88	0.71–1.09	0.88	0.71–1.08	0.82*	0.73–0.93	0.89*	0.79–0.98
**Community economic prosperity**								
Low ^RC^	-	-	-	-	-	-	-	-
Middle	1.10	0.79–1.54	0.68**	0.57–0.80	1.21	0.70–2.10	1.22	0.71–2.08
High	1.17	0.96–1.42	0.60**	0.46–0.74	1.01	0.74–1.38	1.01	0.74–1.36
**Visitation to health facility**								
No ^RC^			-	-			-	-
Yes			0.49**	0.34–0.72			0.76*	0.64–0.91
**Visitation by family planning worker**								
Not visited ^RC^			-	-			-	-
Visited			0.52**	0.35–0.75			0.77**	0.70–0.84
**Health insurance**								
Not enrolled ^RC^			-	-			-	-
Enrolled			1.11	0.91–1.36			1.02	0.47–2.20
ICC (%)	5.4		5.4		15.5		14.8	
AIC	8200.8		8199.5		4479.1		4473.3	

*p < 0.05, **p < 0.01

The likelihood of unmet need for contraception was higher among rural women compared to urban women (aOR = 1.42, 95% CI: 1.16–1.72). The odds of unmet need for contraception were lower among Southern women compared to Northern women (aOR = 0.71, 95% CI: 0.62–0.81). Women who live in communities with a high justification of gender-based violence were more than two times more likely to have an unmet need for contraception compared to women in the reference group (aOR = 2.44, 95% CI: 2.20–2.71). Women who live in communities with high economic prosperity were less likely to have an unmet need for contraception than those in low economic prosperity (aOR = 0.60, 95% CI: 0.46–0.74). Women who visited health facilities within the year were less likely to have an unmet need for contraception compared to those who did not visit health facilities (aOR = 0.49, 95% CI: 0.34–0.72). Likewise, women who were visited by family planning workers had lower odds of unmet need for contraception compared to those not visited (aOR = 0.52, 95% CI: 0.35–0.75). The result of the ICC (ICC = 5.4%) indicated that the community factors had less influence on variations in unmet need for contraception compared to the individual factors.

### Fixed and random effects on unmet need for contraception in high-priority segment

As shown in Table 3 in respect of the high-priority segment. In Model 1 based on the ICC, the variations of unmet need for contraception were moderate (not shown). Model diagnosis based on the AIC indicated that the full model had the best goodness of fit. In Model 2, six individual characteristics, namely, education, work status, religion, desired family size, media exposure, and pregnancy termination were the significant predictive factors of the unmet need for contraception. But at the household level, sexual autonomy was the only characteristic that revealed a significant effect on unmet need for contraception. Place of residence, geographic region, community justification of gender-based violence, and proportion of ever-used contraception in the community were the important predictive factors at the community level. However, the ICC of 15.5% shows that less than one-fifth of variations in unmet need for contraception could be deemed to have been caused by community characteristics. This pattern was also observed in Model 3. In the model, women who attained secondary and higher education had higher odds of unmet need for contraception than uneducated women. Muslim women had a lower likelihood of an unmet need for contraception compared to Christian women (aOR = 0.61, 95% CI: 0.48–0.79). The odds of an unmet need for contraception were lower among women who desired large family sizes compared to women who desired small family sizes (aOR = 0.69, 95% CI: 0.56–0.84). The odds of an unmet need for contraception are reduced as media exposure improves. Likewise, the odds were lower among women who had experienced pregnancy termination (aOR = 0.57, 95% CI: 0.42–0.78).

Sexually autonomous women had lower odds of unmet need for contraception compared to those not sexually autonomous (aOR = 0.63, 95% CI: 0.52–0.75). While rural women were less likely to report an unmet need for contraception (aOR = 0.84, 95% CI: 0.78–0.92), Southern women were more likely to report an unmet need for contraception (aOR = 1.45, 95% CI: 1.09–1.92). The odds of an unmet need for contraception increase as justification of gender-based violence becomes more pronounced in the community. Women who live in communities where a high proportion of women had ever used contraception were less likely to have an unmet need for contraception compared to women in the reference category (aOR = 0.88, 95% CI: 1.01–1.65). Two control variables, namely, visitation to health facilities and visitation by family planning workers reveal significant effects on the likelihood of an unmet need for contraception. The decline in the ICC to 14.8% provided evidence that the community characteristics did not cause much variation in the unmet need for contraception across the communities.

## Discussion

This study examined differences in the prevalence and determinants of unmet need for contraception among women in low and high-priority segments for family planning demand generation in Nigeria. This was done to provide information on the need to close the existing gap in the provision of contraceptive services to all segments of men and women. This is particularly important in Nigeria, where the family planning blueprint designated adolescents, young adults, and unmarried people as high-priority segments for family planning demand generation [6, 49] without sufficient consideration for women in peculiar population groups, such as women in advanced reproductive age group and women living with disabilities. With enough evidence that this latter group of women is usually underserved by contraceptive services across the world [8, 11, 12, 14, 16–18], it is important that family planning programmers take more steps to meet the contraceptive need of everyone irrespective of living conditions. This will not only enhance the attainment of universal access to reproductive healthcare in Nigeria, but will also accelerate the country’s prospects of reaping the demographic dividends [3–5].

The study found that the prevalence of unmet need for contraception is higher among women in the low-priority segment compared to the prevalence among women in the high-priority segment for family planning demand generation in Nigeria. On the one hand, the prevalence observed among women in the low-priority segment is comparable to the 25.2% reported for older women in Botswana [7], and the 22.9% reported for urban women of advanced reproductive age in The Gambia [9]. On the other hand, the prevalence observed among women in the high-priority segment is lower than the overall prevalence of 24.9% and 26.9% reported among adolescents and young adults in two recent studies that focused on sub-Saharan Africa [25, 40]. It is, however higher than the 11.7% observed among adolescents in Cambodia [53]. Despite the differences, the prevalence found among the two segments in the study points to the need for national family planning programmers to pay more attention to the sexual and reproductive health of women in the country by taking additional steps to help women regardless of living conditions to avoid pregnancies when not desired through the use of modern contraceptives.

This will not only reduce the level of unintended pregnancies [29, 31], but it will also minimize unsafe abortion [30], and promote women’s sexual autonomy [60], which further enhances women’s reproductive health. It is particularly important for the national family planning programmers to give special consideration to the plight of women living with disabilities. Studies have provided evidence that in many countries, women living with disabilities face a lot of demand and supply-side barriers to accessing contraceptive services [15], which may have translated to the low use of modern contraceptives, as found in many existing studies among both young and older women living with disabilities [11, 14, 16]. In the case of Nigeria, both the family planning blueprint and the revised population policy [49, 50] do not in any remarkable way mainstream the contraceptive needs of women living with disabilities into the family planning demand generation strategies. The revised population policy merely included people living with disabilities in the list of special population groups. This should be complemented by the design of specific strategies to remove all transportation, education, communication, and other barriers to access to contraceptive services among such women. For instance, creating a particular unit for women living with disabilities within the family planning division in public hospitals may address some of the known barriers.

Findings also reveal that while some of the determinants of unmet need for contraception cut across the two segments, some of the determinants are peculiar to specific segments. This finding may be reasoned in two ways. Firstly, the determinants that cut across and in accordance with existing findings are religion [38], desired family size [36, 42], place of residence [28, 41], geographic region [37, 48], community justification of gender-based violence [47], and lack of sexual autonomy, which further exposes women to adverse sexual health outcomes [60]. This finding implies that some cross-cutting strategies, either at the individual or community level, targeting selected socio-demographic characteristics, may work effectively to reduce the unmet need for contraception among groups of women. This was well-demonstrated in a project implemented at the individual, community, and structural levels to improve family planning and sexual and reproductive health programs in Akwa Ibom State, Nigeria [55]. Community characteristics are particularly important for improving women’s contraceptive behavior because in many instances, the family planning information provided by women’s friends and relatives, or shared within women’s social networks may sometimes override their individual characteristics, and to a large extent influence women’s decision to use or not to use modern contraceptives. This tends to support the need for more community-based family planning initiatives to improve access to modern contraceptive services in the communities.

Though, the context in which a specific socio-demographic characteristic influences contraceptive behavior varies from one group of women to another, family planning programmers in the country must clearly understand what works, and why it works among each group of women. For instance, as found in this study, the desire for a large family size was a significant determinant in the two groups of women. While it is true that the desire for a large family size could lead to a lack of interest in using contraceptives to space or limit pregnancies in the two groups, its pathways might differ in the two groups. It is possible that among young adults in the high-priority group, non-use of contraceptives may be caused by a lack of youth-friendly reproductive health services in the community, in contrast, non-use may likely result from cultural, religious or spousal factors among women in the low-priority group [32].

Secondly, the finding that some determinants are peculiar to specific segments of women suggests that in addition to cross-cutting strategies, there is a tendency that a one-size-fits-all approach may not work optimally in the generation of family planning demand among women. Hence, the series of family planning demand-generation initiatives of the government of Nigeria, such as the development of the National Family Planning Communication Plan (NFPCP), the resuscitation of the National Health Promotion Forum, and the launch of the new FP logo ‘The Green Dot’ [49] should be re-examined to develop specific strategies that will resonate with each segment of reproductive age women. For instance, while family planning messages via the radio and television may appeal more to older women, adolescents and young adults may find family planning messages via new and social media more appealing.

### Strengths and limitations of study

The analyzes of the 2018 NDHS datasets is an important strength of the study because the datasets are not only of high quality, they are also available in the public domain. This provides opportunity to researchers in other climes to verify and replicate the study. The study provides additional support for the socio-ecological theory by confirming that the unmet need for contraception is influenced by multiple factors operating at different levels of the social environment. However, the study could not investigate how the unmet need for contraception, in turn, shapes each of the socio-environmental factors in line with the propositions of the socio-ecological theory. The cross-sectional nature of the data analyzed implies that the regression analysis performed is best understood as connoting a strong correlation of the research variables and not in any way establishing causality between the variables. Also, some variables already identified as predictors of unmet need for contraception are not analyzed in the study if such variables do not cut across the two groups of women examined. It is thus possible that the inclusion of such variables in future analyzes may moderate the findings of the study.

## Conclusion

This study examined differences in the prevalence and determinants of unmet need for contraception among women in low and high-priority segments for family planning demand generation in Nigeria. Based on secondary analyzes of the merged data extracted from individual and persons recode of the 2018 NDHS, it was found that the prevalence of unmet need for contraception differs among women in the low and high-priority segments. Furthermore, evidence reveals that some determinants of unmet need for contraception cut across both segments of women, while some are peculiar to a specific segment of women. This finding suggests that despite the importance of cross-cutting strategies to reduce unmet need for contraception among women, there is a need to equally recognize the peculiarities of each segment, and tailor strategies to specific segments in terms of family planning demand generation in Nigeria.

## Data Availability

The dataset analyzed is available in the public domain. Interested persons could access it online at https://dhsprogram.com/data/dataset/Nigeria_Standard-DHS_2018.cfm?flag=1.

## Abbreviations

DHS: Demographic and Health Survey
FMoH: Federal Ministry of Health
ICF: Inner-City Fund
NDHS: Nigeria Demographic and Health Survey
NPC: National Population Commission
WHO: World Health Organization
UN: United Nations
USAID: United States Agency for International Development
